# Fault-Finding

**Published:** 1872-09

**Authors:** 


### FAULT-FINDING.

Whole oceans of heart-aches, and passions as fierce as the fabled fires of perdition, if indeed oceans could be measured with a tin ladle, could be prevented every year in the parlor, the family room, at the breakfast table, at the store, the bank, the manufactory, if one single proper, practicable, and rational rule could be observed all over the face of the globe for one single day. Never find fault with anybody, never blame anybody, never scold anybody, in the presence of a third person. If I can induce any considerable number of persons to put in practice this rule, from the instant, to be continued to the end of life, I will have lived to greater purpose than all the Alexanders and Cæsars and Napoleons of past ages. If one single human being, a father, or a mother, should make and carry out such a resolution, my life will not have been a useless one. I shall go to my bed every night, and down into my grave when all my nights are ended, the happier at the thought that some little child's feelings have not been hurt; some husband, some wife, some worthy servant, has been made happier than if I had never lived. If you must blame anybody, and you want to do it most efficiently, let it be when you two are alone. A nice, manly little youth of fourteen, committed suicide the other day in Brooklyn, because, having committed a fault, he feared to face his stern father, whose habit was, in order to make his criticism more impressive, to have all the members of the family present. He left a little scrawl saying, "Johnny won't trouble you all any more; good-by!"

